# Synthesis of Rh-MOF/PVA-PVP nanofibers for skin cancer and infection inhibition

**DOI:** 10.3389/fchem.2025.1575183

**Published:** 2025-04-28

**Authors:** Ali Altharawi, Taibah Aldakhil, Manal A. Alossaimi

**Affiliations:** Department of Pharmaceutical Chemistry, College of Pharmacy, Prince Sattam Bin Abdulaziz University, Al-Kharj, Saudi Arabia

**Keywords:** rhodium, metal-organic framework, nanofibers, anti-skin cancer, anti-skin infections

## Abstract

Using electrospinning for nanofiber production, we can create unique materials with multiple applications in various industries, including medical bandages and wound dressings. One of the most important features of these materials and using the electrospinning technique, is the incorporation of compounds and metals into their structure. In this study, a new metal-organic framework (MOF) was synthesized from rhodium, a metal with significant biological potential, which was then used to produce new nanofibers using electrospinning technique, (Rh-MOF/PVA-PVP nanofiber) by mixing polyvinyl alcohol (PVA) and polyvinylpyrrolidone (PVP). The newly synthesized nanofiber was tested against common microbial skin pathogens and cancer cells, showing significant inhibition. Specifically, an IC_50_ value of 19.45 μg/mL against cancer cells and MIC values ranging from 4 μg/mL to 64 μg/mL μg against skin pathogenic strains were observed. This notable inhibitory ability can be attributed to both physical characteristics (with specific surface area of 2,348 m^2^/g), and chemical factors, including the active compounds present in its rhodium (Rh) structure. The synthesized Rh-MOF/PVA-PVP nanofiber has the potential for use in developing bioactive bandages, and wound dressings.

## 1 Introduction

Rhodium, one of the rarest and most expensive metals in the periodic table, captivates attention with its extraordinary properties across various fields (Jia M. et al., 2022). This remarkable metal not only serves as a powerful catalyst in chemical reactions (Ouyang et al., 2020), but it also plays a crucial role in photocatalysis (Yang and Ohtani, 2021). Additionally, rhodium exhibits, hydroformylase activating enzyme (Jarvis et al., 2017), anti-cancer properties (Máliková et al., 2021), and antimicrobial effects (Tan et al., 2023), making it a truly versatile element.

Numerous complexes and nanostructures containing rhodium have been reported in the literature, such as Rh-M (M = Fe, Ru, Mn, Rh, Pt, Pd, Re, Cu, Ni)-BTC-metal-organic framework (MOF) (Chen et al., 2023), monophosphine Rh complexes by Zr-MOF (Zheng et al., 2024), and Rh@MIL-53(Al) MOF (Isaeva et al., 2025). A recent study demonstrated that a newly developed rhodium-containing MOF has the potential to absorb nitrogen oxides (NO gas) (Hu et al., 2023). One of the main reasons for the high nitrogen gas absorption activity of this composition is its metal-organic framework (MOF) structure, which provides a high specific surface area and tunable porosity (Hu et al., 2023).

Metal-organic frameworks are materials composed of metal ions coordinated to organic ligands, forming a porous structure with unique properties (Cai et al., 2021). Due to their high specific surface area, MOFs have attracted significant attention from scientists and researchers for various applications, including gas storage (Jia T. et al., 2022), separation processes (Deng et al., 2021), catalysis (Shen et al., 2021), and drug delivery (Lawson et al., 2021). Their versatility allows them to be engineered for specific functions in environmental science (Lopez et al., 2021), energy storage (Sandhu et al., 2024), and biomedicine (Haider et al., 2022).

One of the most notable applications of MOFs is in biomedicine, particularly in the development of advanced materials for wound healing (Bigham et al., 2024). Fibers made from natural polymers such as polyvinyl alcohol (PVA) (Panda et al., 2022), polyvinylpyrrolidone (PVP) (Belgibayeva et al., 2023), and chitosan (Ilyas et al., 2022) can incorporate various composition, including, nanostructures and plant extracts, through coordination interaction, as well as, multiple hydrogen bonds (Mahmud, 2023). These fibers exhibit relatively high compressive and flexural strength, making them suitable for use as wound dressings and medical bandages (Nguyen et al., 2023).

The inclusion of biologically active compounds in these fibers has garnered much attention due to their potential therapeutic effects (Adamu et al., 2021). Among the biological properties reported for these fibers are antimicrobial (Maliszewska and Czapka, 2022; Torres-Cortés et al., 2022) and anticancer activities (Zhao and Cui, 2020). Since wound dressings and bandages are in direct contact with the skin, they can be crucial in treating infectious and cancerous skin diseases (Farahani and Shafiee, 2021). The most common infectious skin diseases include cellulitis, impetigo, folliculitis, boils and carbuncles, erysipelas, and erythrasma (Mercieca and Pace, 2022; Bessa, 2023). Common bacterial agents responsible for these diseases include *Streptococcus pyogenes, Streptococcus dysgalactiae subsp. equisimilis, Streptococcus anginosus, Staphylococcus aureus, Pseudomonas aeruginosa, and Corynebacterium minutissimum* (Citron et al., 2007; De la Maza et al., 2020). So far, no fiber compounds have been reported that are specifically effective against bacterial skin diseases as well as anticancer activity.

Given rhodium’s anticancer and antimicrobial properties, this precious metal has the potential to inhibit both skin cancer cells and pathogenic bacterial strains responsible for skin infections. Therefore, a new fiber can be synthesized by focusing on these capabilities. A novel MOF structure containing rhodium was synthesized as a compound with antibacterial and anticancer properties. This MOF was then utilized in the production of new fibers using the electrophoresis techinique with PVA and PVP. The newly produced fibers were tested for antimicrobial properties against common microbes associated with skin infections. Preliminary results indicated promising antibacterial activity against several strains of bacteria known to cause skin infections. This suggests that rhodium-containing fibers could serve as effective wound dressings or medical bandages with dual functionality: promoting healing while preventing infection.

Rhodium’s unique properties and its incorporation into metal-organic frameworks (MOFs) present exciting opportunities for developing advanced materials with significant biomedical applications. The synthesis of rhodium-containing fibers not only enhances their mechanical properties but also imparts valuable biological functions that could improve patient outcomes in wound care. This version expands on the original content by providing more detailed explanations about MOFs, their applications in biomedicine, and the potential benefits of rhodium-containing materials.

In summary, the synthesis of a new rhodium-containing morph, the production of a new fiber with environmentally friendly polymers, and a specialized study on skin diseases for its practical use in the production of wound dressings and medical bandages are some of the novelties of this study compared to recent reports.

Providing insights into how rhodium interacts with biological targets (e.g., reactive oxygen species generation, DNA damage, or membrane disruption), and *in vitro* or *in vivo* wound healing assays can be suggested as further and complementary studies to this study.

## 2 Experimental

### 2.1 Materials, devices, characterization

#### 2.1.1 Materials

Rhodium (III) nitrate was sourced from Nanochemazone, a reputable supplier known for high-purity chemicals (99.9%).

The 2,2′-bipyridine-4,4′-dicarboxylic acid (98%) was obtained from Sigma-Aldrich, which specializes in fine chemicals and reagents. These materials are commonly used in various chemical syntheses and research applications.

The bacterial strains and cancer cells used in this study were obtained from the American Type Culture Collection (ATCC). ATCC offers a diverse selection of well-characterized cell lines, including those relevant to cancer research. These cell lines are crucial for investigating various facets of cancer biology, such as tumor development, treatment responses, and the molecular mechanisms underlying cancer progression.

#### 2.1.2 Devices

The MOFs were synthesized using an BP110 laboratory grade microwave (power of 325 W and at room temperature). The nanofibers were produced using an NE100 Single Nozzle Electrospinning Machine (flow rate of 0.4 mL/h, a needle-to-collector distance of 22 cm, and an applied voltage of 28 kV).

#### 2.1.3 Characterization

To confirm the structure and properties of the synthesized products, several analytical techniques were employed:

- FT-IR Spectroscopy: Used to identify functional groups and confirm molecular structures (Thermo AVATAR - ATR-FTIR).
- Scanning Electron Microscopy (SEM): Provides images of the surface morphology (Hitachi S-4800; 102 Pa, 15 kV, 150 kX)
- CHNO Elemental Analysis: Determines the composition of carbon, hydrogen, nitrogen, and oxygen in the samples (Thermo EA1112; The sample is broken down via combustion in an oxygen atmosphere at 1,000°C).
- X-Ray Diffraction (XRD): Analyzes crystal structures and phase purity of the synthesized materials (shimadzu 7000; Cu-Kα radiation (λ = 1.5418 Å), 10–80°, rate of 1.5°/min.).
- Nitrogen Adsorption/Desorption Isotherms: Measures surface area (BELSORP mini II).
- UV-Vis spectrophotometer: Used to Preparation of bacterial strain suspension concentrations (Perkinr- UV-VIS pectrum RX1).
- Flexural strength and Compressive strength using NanoBionix, MTS (25°C, humidity of 40%)

Inverted microscope and ELISA reader: To investigate anticancer activities (TCM-400 inverted microscope, and BioTek-ELx800 ELISA reader).

### 2.2 Synthesis

#### 2.2.1 Rh-MOF

The synthesis of rhodium-based metal-organic frameworks (MOFs) was carried out using methods previously reported for similar MOFs (Bashar et al., 2022; Saadh et al., 2024). In this process, 325 mg of rhodium (III) nitrate and 167 mg of 2,2′-bipyridine-4,4′-dicarboxylic acid were dissolved in 20 mL of deionized water (in 50 mL bottom flask). Then, it was stirred at 80 °C for 15 min carried out on hot plate to ensure proper mixing and dissolution. Following this, the homogenized solution was subjected to microwave radiation at a power of 325 W and a room temperature for an additional 15 min to promote the formation of the Rh-MOF.

After the synthesis, the resulting rhodium metal-organic framework (Rh-MOF) was isolated through nanofiltration (using Whatman filter paper) to remove any unreacted materials and byproducts. The synthesized product was then washed three times with a 1:1 mixture of deionized water and ethanol to purify it. Finally, to achieve complete dryness, the product was placed under vacuum at ambient temperature for 48 h.

#### 2.2.2 Rh-MOF/PVA-PVP nanofiber

In 50 mL bottom flask, a solution (20 mL) of polyvinyl alcohol (PVA) and polyvinylpyrrolidone (PVP) in a 1:1 ratio using acetic acid was prepared, resulting in a concentration of 0.004%. Separately, a suspension containing 0.01 mg of the Rh-MOF was created by dispersing it in 25 mL of deionized water (in 100 mL bottom flask). The two solutions were then combined and stirred for 15 min at 80°C to ensure thorough mixing.

Following this, electrospinning was conducted with the following parameters: a flow rate of 0.4 mL/h, a needle-to-collector distance of 22 cm, and an applied voltage of 28 kV. After the solvents (deionized water and acetic acid) evaporated at ambient temperature, Rh-MOF/PVA-PVP nanofiber were successfully synthesized (Moghaddam-manesh et al., 2022; Asiri et al., 2023).

### 2.3 Biological

#### 2.3.1 Antimicrobial

The antibacterial activity of the tested compounds was assessed using methods established by the Clinical and Laboratory Standards Institute (CLSI). Bacterial strains were prepared at a concentration of 1 × 10^5^ CFU/mL in Mueller Hinton broth, which is a standard medium used for antimicrobial susceptibility testing. This study aimed to determine both the Minimum Inhibitory Concentration (MIC) and the Minimum Bactericidal Concentration (MBC) through microdilution methods and kill assays (Ahani et al., 2018; Altharawi et al., 2024; Saadh et al., 2024).

#### 2.3.2 Anticancer

The anticancer activity of the synthesized Rh-MOF/PVA-PVP nanofiber, was evaluated using non-radioactive cell proliferation assay protocols, specifically the MTT method (Moghaddam-Manesh and Hosseinzadegan, 2021; Altharawi et al., 2024; Saadh et al., 2024). Skin cancer cells were treated with varying concentrations (6.25, 12.5, 25, and 50 μg/mL) of the synthesized hydrogel for periods of 24 and 48 h. To ensure reliability, the anticancer activity analyses were repeated three times, and the reported results represent the average of these three replicates.

In this study, skin cancer cells were cultured to a density of 1.2 × 10^4^ cells and treated with the specified concentrations of Rh-MOF/PVA-PVP nanofiber. The cell culture medium used was RPMI 1640 (Roswell Park Memorial Institute) supplemented with 10% Fetal Bovine Serum (FBS) and antibiotics, including gentamicin and penicillin. The incubation conditions were maintained at 37°C in a humidified atmosphere containing 5% CO2.

After treatment with the hydrogel nanopolymer, MTT solution was added to each well, followed by incubation for an additional 4 hours. This allowed viable cells to convert the MTT into formazan crystals. Subsequently, diethyl sulfoxide (DMSO) was added to dissolve these crystals, and the absorbance was measured at 570 nm using an ELISA reader to determine cell proliferation and viability in response to Rh-MOF/PVA-PVP nanofiber.

## 3 Result and discussion

### 3.1 Synthesis findings result and discussion

Rhodium-metal-organic frameworks (Rh-MOF) were synthesized through the reaction of rhodium, which has been noted in previous studies for its biological properties (Dai et al., 2023), and a 2,2′-bipyridine-4,4′-dicarboxylic acid with two carboxylic acid groups, known for its biological properties due to the presence of two pyridine rings in its structure (Mahadevi et al., 2022). The synthesis was carried out using the microwave method, which is a suitable and validated approach for the preparation of MOFs ((Gao et al., 2024). The optimal conditions included power of 325 w and a temperature of 25°C, which previous reports have shown to result in products with suitable properties (Bashar et al., 2022; Saadh et al., 2024).

This microwave-assisted synthesis method enhances reaction rates and improves yields compared to traditional heating techniques, potentially resulting in Rh-MOF with unique properties suitable for applications such as biological activity, including higher stability and synthesis of composition with higher specific surface area (Gangu et al., 2022).

**FIGURE 1:**
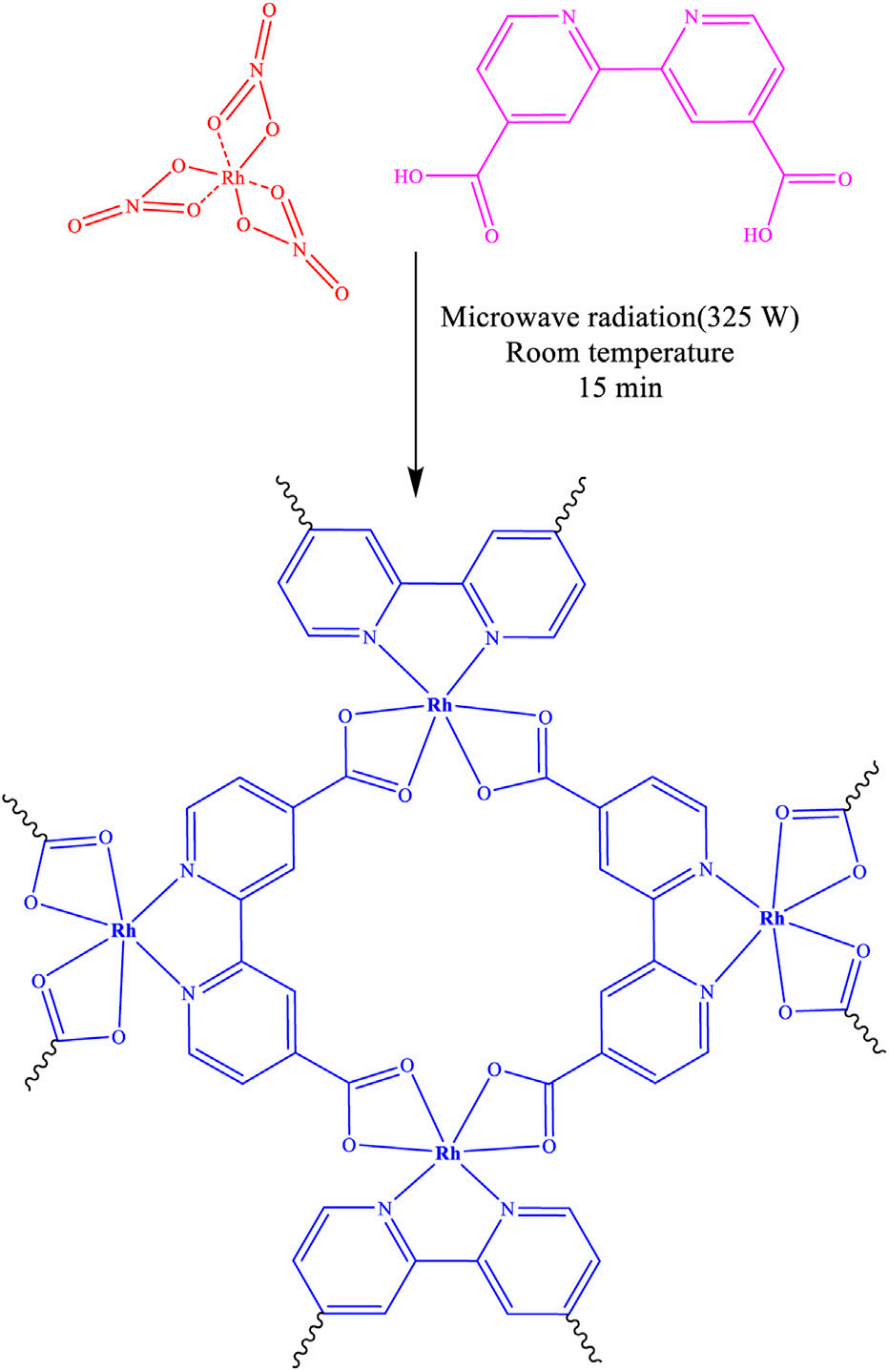
Synthesis of Rh-MOF.

The structure of the final product is illustrated in Figure 1 and was subsequently confirmed and characterized using FT-IR, XRD, EDAX, EA, SEM, and BET.

2,2′-Bipyridine-4,4′-dicarboxylic acid has the can form complexes with Rhodium (III) nitrate under microwave conditions through the coordination of the carboxylic acid oxygens and pyridine nitrogens.

**FIGURE 2:**
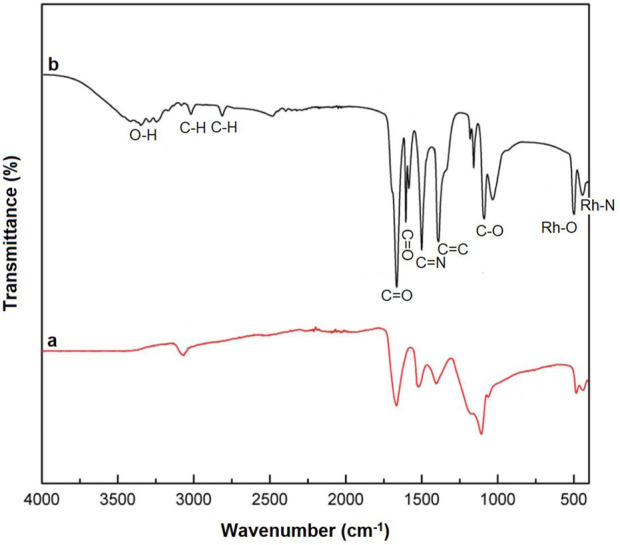
FT-IR of Rh-MOF **(a)** and Rh-MOF/PVA-PVP nanofiber **(b)**.

As seen in its FT-IR spectrum (Figure 2a), Rh-N bonds (bending in the plain) were observed in the region around 460 cm^−1^ (Abdulameer and Alias, 2022), while Rh-O bonds (bending in the plain) were detected in the region around 498 cm^−1^ (Khorshidi and Sadeghi, 2016).

Other peaks that confirmed the presence of the ligand in the final product included C-O, C=C, C=N, C=O, and aromatic C-H bonds, which were observed in regions 1,130 cm^−1^, 1,435 cm^−1^, 1,540 cm^−1^, 1,620 cm^−1^, and 3,045 cm^−1^, respectively. 2,2′-Bipyridine-4,4′-dicarboxylic acid has two carboxylic acid groups (COOH), which produce a broad peak in the region of 3,200–3,500 cm^−1^ (O-H). The absence of these peaks in the final product (COOH), along with the presence of the Rh-O peak, serves as evidence for the complexation of 2,2′-Bipyridine-4,4′-dicarboxylic acid through its oxygen atoms.

**FIGURE 3:**
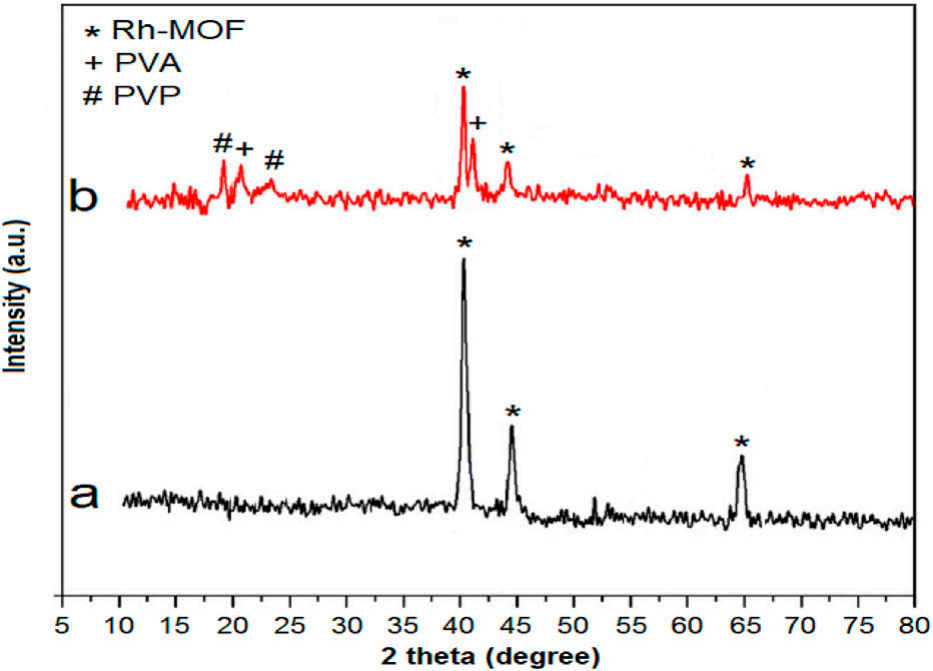
XRD of Rh-MOF **(a)** and Rh-MOF/PVA-PVP nanofiber **(b)**.

The XRD analysis of the Rh-MOF shown in Figure 3a reveals peaks at 41.1° (111), 44.6° (200), and 64.7° (220). These peaks correspond to JCPDS 05-0685, which is associated with the octahedral crystal structure of Rh-MOF (Zhang et al., 2016).

**FIGURE 4:**
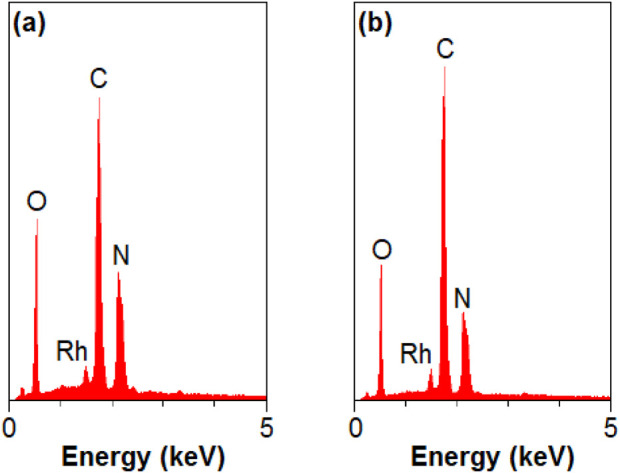
EDAX of Rh-MOF **(a)** and Rh-MOF/PVA-PVP nanofiber **(b)**.

The EDAX and EA of the Rh-MOF, as shown in Figure 4a and detailed in Table 1-a, confirm the presence of radium, carbon, hydrogen, nitrogen, and oxygen in the Rh-MOF.

**TABLE 1:** EA of Rh-MOF (a) and Rh-MOF/PVA-PVP nanofiber (b).

Compound	Element			
	% C	% H	% N	% O
a	45.12	3.38	7.06	16.56
b	57.43	6.55	8.98	17.71

Therefore, based on the information above, the synthesized Rh-MOF is consistent with and confirmed by the structure reported in Figure 1.

Other characterizations of the Rh-MOF, such as those discussed in SEM, BET, and TGA, are presented after confirming the initial structure of the nanofiber with FT-IR, XRD, EDAX, and EA for comparison.

One method for producing fibers involves the use of PVA and PVP, which are both commonly used in fiber production.

**FIGURE 5:**
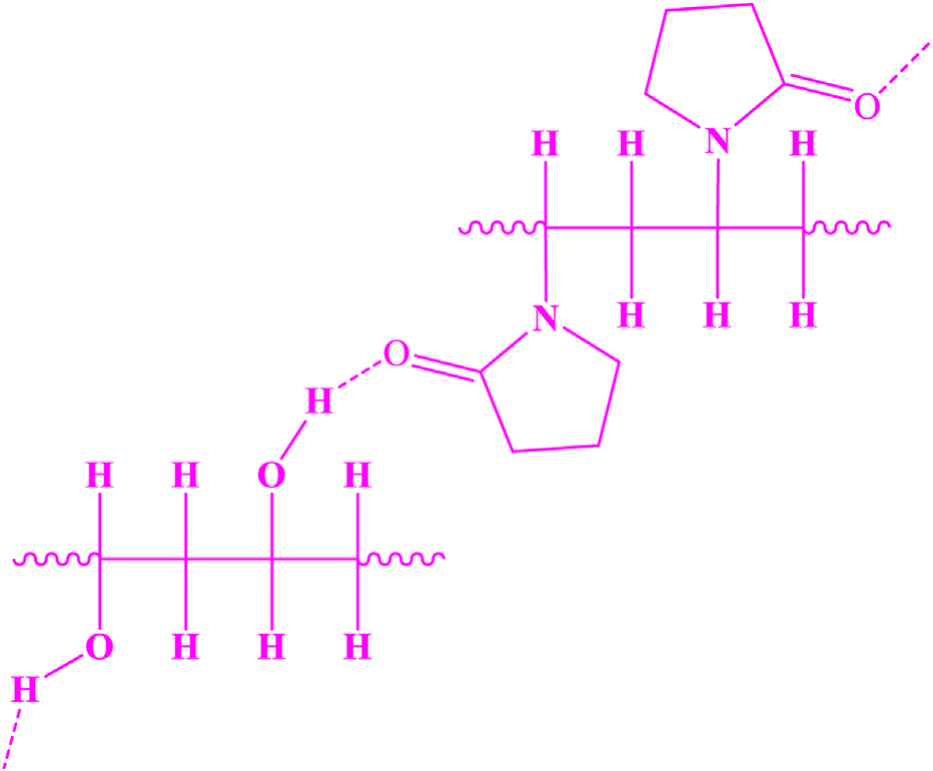
Structure of PVA-PVP.

These two compounds can form hydrogen bonds, resulting in the structure shown in Figure 5.

Since the mixture of PVA and PVP results in a polymer with enhanced hydrogen bonding capabilities, and previous studies have shown that this mixture provides unique physical properties superior to those of either polymer alone, such as a higher specific surface area, it was used in fiber production.

**FIGURE 6:**
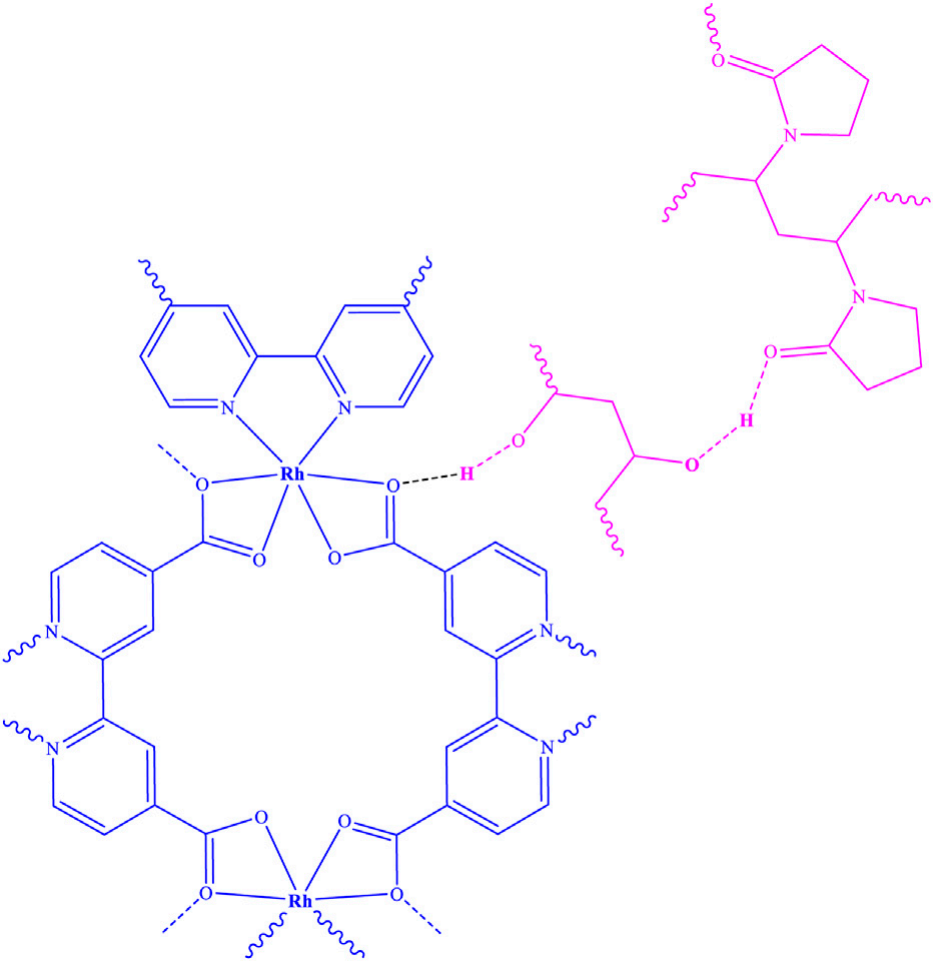
Structure of Rh-MOF/PVA-PVP nanofiber.

As a result of the electrospinning reaction between the Rh-MOF and PVA-PVP nanofiber, along with the formation of hydrogen bonds between them, a new fiber was synthesized (Rh-MOF/PVA-PVP nanofiber). Figure 6 illustrates this new fiber, and its proposed structure has been confirmed and characterized by FT-IR, XRD, EDAX, EA, SEM, BET, flexural strength, compressive strength, and hydrophilicity (contact angle) (Asiri et al., 2023). From the green benefits of this study is the use of environmentally friendly polymers, namely PVA and PVP, in the production of nanofibers.

The Rh-MOF/PVA-PVP nanofibers exhibit FT-IR peaks similar to those of Rh-MOF, with only minor differences between them (Figure 2b). The observed difference is the O-H peak in region 3,300 cm^−1^ (related to PVA), and aliphatic C-H peak in region 2,850 cm^−1^ (related to PVA and PVP) of the Rh-MOF/PVA-PVP nanofiber FT-IR spectrum. Another difference is the presence of two carbonyl peaks (C=O, related to PVP and Rh-MOF) in the 1,600–1,650 cm^−1^ range, and two C-O peaks in the 1,000–1,130 cm^−1^ range, corresponding to the Rh-MOF and the PVP-PVA.

The comparison of the XRD patterns of Rh-MOF and Rh-MOF/PVA-PVP nanofiber (Figure 3b) shows that more peaks were observed at angles 19.2°, 20.7°, 23.6°, and 41.8°. Based on previous studies, the angles 20.7° and 41.3° correspond to PVA (Ricciardi et al., 2004), and the angles 19.2° and 23.6° correspond to PVP (Benmore et al., 2023).

The EDAX and EA of the Rh-MOF/PVA-PVP nanofiber, as shown in Figure 4b and detailed in Table 1-b, confirm the presence of radium, carbon, hydrogen, nitrogen, and oxygen in the Rh-MOF.

Comparing the percentages of elemental components in the EA of the Rh-MOF and the Rh-MOF/PVA-PVP nanofiber shows that the percentage of elements, mainly carbon, and hydrogen, are higher in the Rh-MOF/PVA-PVP nanofiber than in the Rh-MOF, confirming the presence of PVA and PVP in the final product.

**FIGURE 7:**
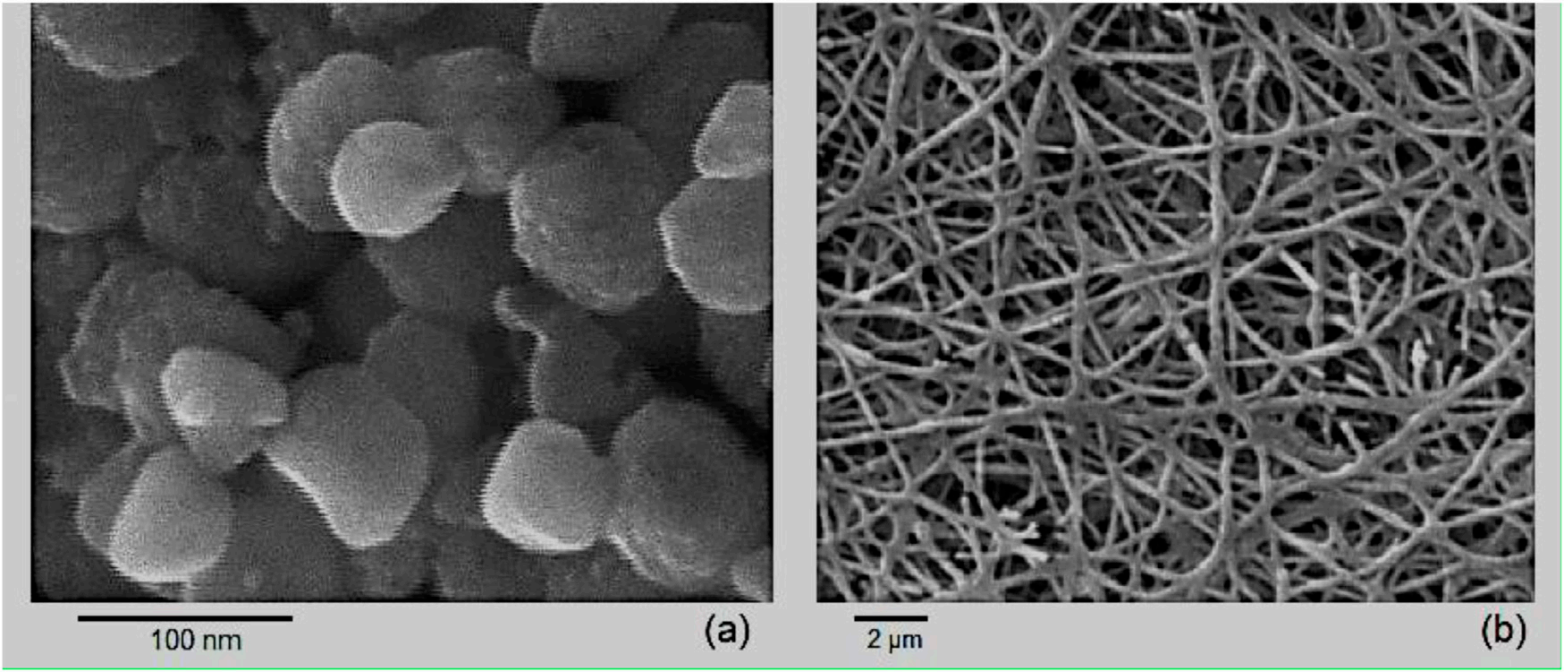
SEM of Rh-MOF **(a)** and Rh-MOF/PVA-PVP nanofiber **(b)**.

The SEM images of the Rh-MOF (a) and the Rh-MOF/PVA-PVP nanofiber (b), as shown in Figure 7, confirm that both exhibit similar morphology and that these synthetic compounds are in the nanoscale range.

**FIGURE 8:**
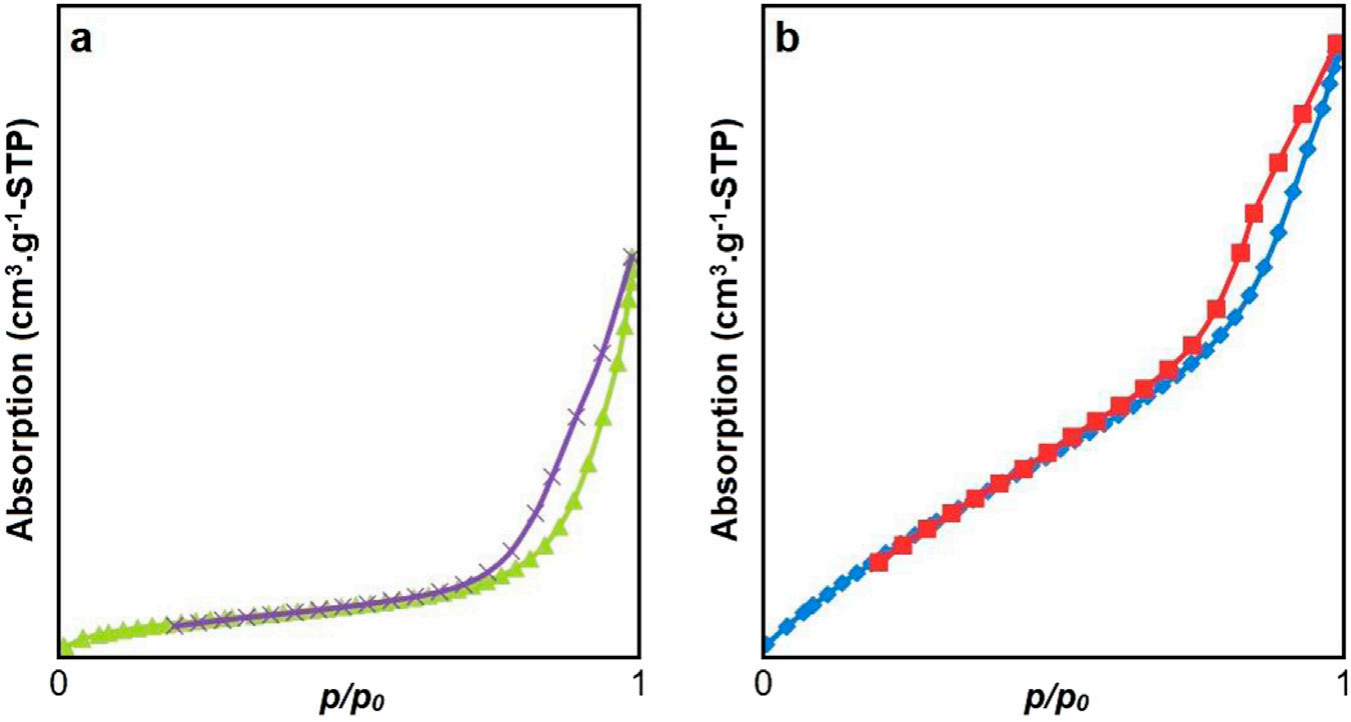
Nitrogen adsorption/desorption isotherms of Rh-MOF **(a)** and Rh-MOF/PVA-PVP nanofiber **(b)**.

The specific surface area, based on nitrogen adsorption/desorption isotherms, for Rh-MOF and the Rh-MOF/PVA-PVP nanofiber, was measured to be 1,589 m^2^/g (type III isotherm) and 2,348 m^2^/g (type IV isotherm), respectively (Figure 8). Rh-MOF/PVA-PVP nanofiber exhibits mesoporous material behavior (Al-Ghouti and Da’ana, 2020). The higher specific surface area of the Rh-MOF/PVA-PVP nanofiber compared to the Rh-MOF can be attributed to the more significant number of hydrogen bonds present in its structure (Shui et al., 2024). In porous or nanostructured materials, increased hydrogen bonding can promote the formation of intricate networks or layers, which may increase the specific active surface area by creating more accessible surfaces (Liang et al., 2009; Van der Lubbe and Fonseca Guerra, 2019).

From the results obtained, it can be concluded that a crystalline compound has been synthesized in the nanoscale range with a specific surface area. The structural features of these nanocompounds have a direct relationship with their synthesis method; therefore, appropriate methods were employed in their synthesis (Gong et al., 2023).

Other complementary analyses specific to fibers, such as flexural strength, compressive strength, and hydrophilicity, were conducted to characterize the synthesized Rh-MOF/PVA-PVP nanofiber in this study, which are discussed below.

**FIGURE 9:**
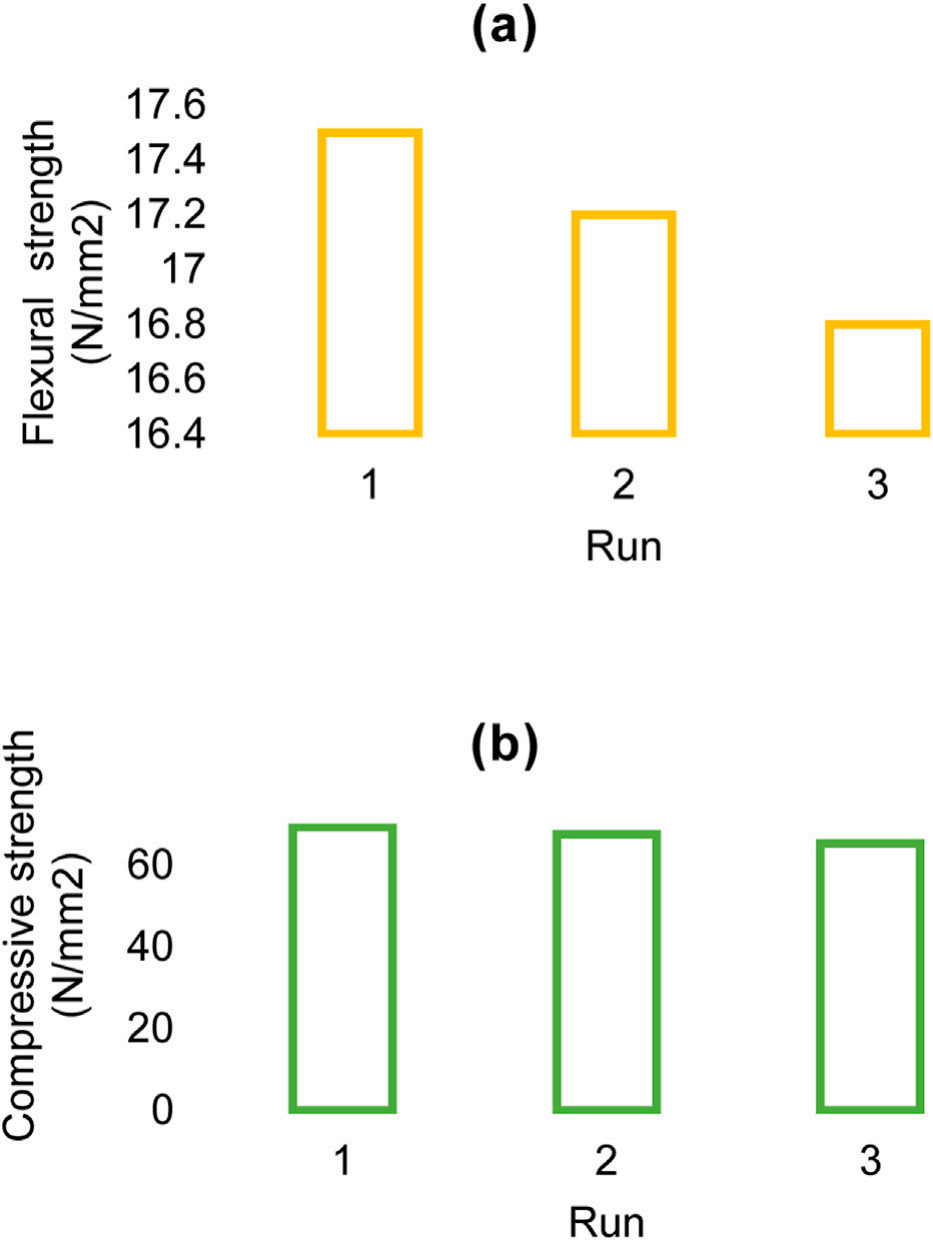
Flexural strength **(a)** and compressive strength **(b)** of Rh-MOF/PVA-PVP nanofiber.

The flexural strength (a) and compressive strength (b) of the Rh-MOF/PVA-PVP nanofiber were measured at 17.2 N/mm^2^ and 67.3 N/mm^2^, respectively (Figure 9). When comparing the Rh-MOF/PVA-PVP nanofiber to other similar compounds, it was found that the fiber synthesized in this study exhibits higher flexural and compressive strengths (Asiri et al., 2023), which can be attributed to the presence of PVA and PVP with multiple hydrogen bonds in the final product.

**FIGURE 10:**
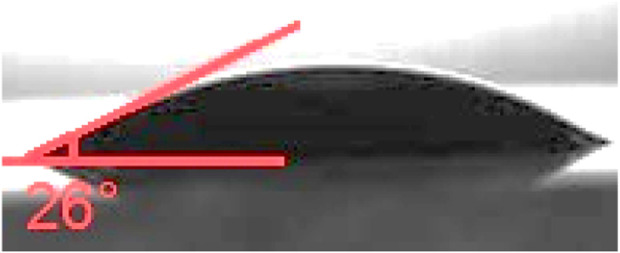
Contact angle of Rh-MOF/PVA-PVP nanofiber.

The contact angle (Figure 10) test of the final product, which measures the hydrophilicity of the fibers, was conducted in this study. The contact angle of the Rh-MOF/PVA-PVP nanofiber was measured at 26°, while the contact angles of PVA and polyvinyl pyrrolidone PVP were reported as 35° (Ngadiman et al., 2015) and 52° (Muzammil et al., 2023), respectively. The high hydrophilicity of the fi Rh-MOF/PVA-PVP nanofiber, confirmed by its lower contact angle compared to PVA and PVP, is attributed to more hydrogen bonding sites than those found in PVA and PVP alone.

### 3.2 Biological findings result and discussion

The use of new technologies in the treatment of diseases and biomedical applications is revolutionizing healthcare, offering innovative solutions to previously complex challenges (Thacharodi et al., 2024). Nanocomposites, which combine a matrix with nano-sized fillers, offer unique properties such as enhanced biocompatibility, controlled drug release, and targeted delivery. These features reduce systemic side effects and improve treatment outcomes. Materials like Metal-Organic Frameworks (MOFs) are highly effective in medical applications due to their high specific surface area, which facilitates greater interaction between active compounds and target agents. This property is particularly useful for treating bacterial infections and cancerous skin diseases (Tripathy and Gupta, 2024). Nanocomposites and MOFs have been extensively studied for their antibacterial properties. They disrupt microbial cells effectively, making them suitable for treating bacterial skin infections and reducing the risk of antibiotic resistance. On the other hand, nanotechnology enables precise targeting of cancer cells through drug delivery systems such as nanoparticles and nano-liposomes. These systems ensure controlled drug release at tumor sites, enhancing treatment efficacy while minimizing damage to healthy tissues (Hu et al., 2024). Nanocomposites provide a platform for delivering therapeutic agents with high specificity, stability, and sensitivity. This is especially beneficial for diseases like bone cancer, where precise drug delivery is critical (Haq Khan et al., 2023). Additionally, nanocomposites mimic the extracellular matrix, supporting tissue regeneration and cell growth in regenerative medicine (Vahidi et al., 2024). Lab-on-nanoparticle devices also utilize nanoscale materials for real-time monitoring and personalized treatment of conditions such as cancer and infectious diseases. Despite their potential, challenges such as scaling up production, ensuring biocompatibility, addressing toxicity concerns, and optimizing degradation rates to meet medical needs remain (Singh et al., 2024). Bridging the gap between materials science and clinical applications is essential for advancing these technologies (Ighodaro et al., 2024).

As we know, one of the applications of fibers that have been industrialized is their use in bandages and wound dressings (Leong et al., 2023). Therefore, if a fiber possesses properties suitable for treating skin infectious diseases and skin cancer, it represents a necessary combination.

The presence of compounds with biological properties (Balewski et al., 2021) and a high specific surface area are two key characteristics of nanoparticles with significant biological activity (Joudeh and Linke, 2022). The synthesized Rh-MOF/PVA-PVP nanofiber in this study contains rhodium and ligands that have been reported to exhibit antibacterial and anticancer properties (Sawkmie et al., 2023). Additionally, its high specific active surface area contributes to its unique biological properties.

Consequently, the antimicrobial effects of the synthesized nanofiber were investigated, particularly against known bacterial strains associated with skin infections, as well as its effectiveness in inhibiting and destroying skin cancer cells.

#### 3.2.1 Antimicrobial findings

Group A *streptococci* (ATCC 19615-*S. pyogenes,* ATCC 12394-*S. dysgalactiae subsp. equisimilis*, and ATCC 33397-*S. anginosus*), ATCC 23235-*Staphylococcus aureus,* ATCC 15442-*Pseudomonas aeruginosa*, and ATCC 23348-*Corynebacterium minutissimum* are known to be the most common bacterial agents of skin diseases, and some common antibiotics are unable to inhibit them. In this study, the minimum inhibitory concentration (MIC) and minimum bactericidal concentration (MBC) of the synthesized Rh-MOF/PVA-PVP nanofiber were evaluated against these bacteria, and tests were also performed to compare their effectiveness with two types of antibiotics (Table 2).

**TABLE 2:** Antimicrobial activity of Rh-MOF/PVA-PVP nanofiber.

Strains	Rh-MOF/PVA-PVP nanofiber		Standard antibiotics			
			Amoxicillin		Erythromycin	
	MIC μg/mL	MBC μg/mL	MIC μg/mL	MBC μg/mL	MIC μg/mL	MBC μg/mL
*S. pyogenes*	8	16	-	-	4	8
*S. dysgalactiae subsp. equisimilis*	64	64	-	-	32	64
*S. anginosus*	16	32	-	-	16	32
*Staphylococcus aureus*	32	64	-	-	4	8
*Pseudomonas aeruginosa*	64	128	-	-	32	64
*Corynebacterium minutissimum*	4	8	-	-	8	16

Surprisingly, the synthesized Rh-MOF/PVA-PVP nanofiber was effective against all the studied strains, resulting in their inhibition. The MICs were observed for *S. pyogenes, S. dysgalactiae subsp. equisimilis*, and *S. anginosus*, *Staphylococcus aureus, Pseudomonas aeruginosa*, and *Corynebacterium minutissimum* as 8 μg/mL, 64 μg/mL, 16 μg/mL, 32 μg/mL, 64 μg/mL and 4 μg/mL, respectively.

The antimicrobial efficacy of MOF compounds is influenced by several structural and physical factors, including their shape, size, and specific surface area (Borsagli et al., 2019; Li et al., 2021; Ding et al., 2024). The porous nature of MOFs, along with their unique hole structures, allows them to effectively trap microorganisms (Arunkumar et al., 2024; Sharafudheen et al., 2024). Additionally, the high specific surface area of MOFs enhances their interaction with bacterial cells, improving their antibacterial performance (Hamarawf, 2024; Perveen et al., 2024).

As previously mentioned, the presence of rhodium and ligand, both of which have biological properties (Mahadevi et al., 2022; Sawkmie et al., 2023), as well as the specific surface area, increases the contact between the Rh-MOF/PVA-PVP nanofiber and bacterial agents (Fahimirad and Shafiee, 2021), can be considered factors contributing to its high antibacterial properties in inhibiting the specialized skin pathogenic strains studied. Notably, the strains studied strains were resistant to Amoxicillin.

#### 3.2.2 Anticancer findings

**FIGURE 11:**
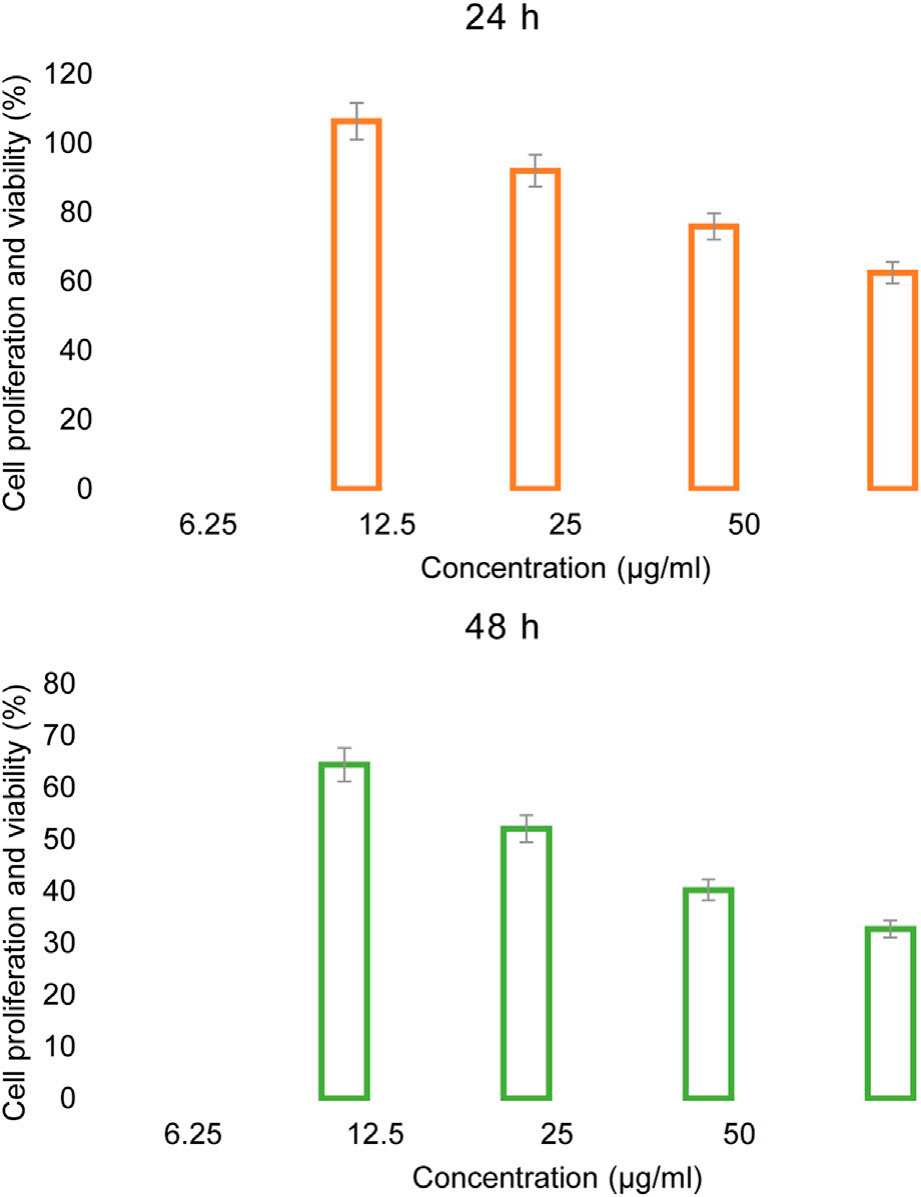
Anticancer activity of Rh-MOF/PVA-PVP nanofiber.

The skin cancer cell studied in this research was ATCC A-431-CRL-1555. First, the cell proliferation and viability than control was assessed at 24 h and 48 h using concentrations of 6.25 μg/mL, 12.5 μg/mL, 25 μg/mL and 50 μg/mL of Rh-MOF/PVA-PVP nanofiber, as shown in Figure 11.

The IC_50_ values calculated for 24 h and 48 h were found to be 59.84 μg/mL and 19.45 μg/mL, respectively.

The results indicated that the highest effectiveness of the Rh-MOF/PVA-PVP nanofiber occurred at 48 h with a concentration of 50 μg/mL, achieving a cell proliferation and viability than control 32.74%.

Based on the obtained results, statistical analyses were performed, as shown in Table 3. As we know, P value indicates the effect of a factor (concentration and exposure time) on the response and if its value is less than 0.05, it indicates that factor has a significant effect on our response. Based on the results, it is confirmed at both concentration and exposure time that the factors have a significant effect on the response (Suksatan et al., 2022).

**TABLE 3:** Statistical analyses.

Time (h)	P value
24	0.002
48	0.001

Table 3 was demonstrated that the effectiveness of the Rh-MOF/PVA-PVP nanofiber on skin cancer depends on both concentration and exposure time; with increasing concentration and duration, greater efficacy and inhibition were observed due to enhanced contact between the Rh-MOF/PVA-PVP nanofiber and cancer cells (Fahimirad et al., 2021).

Regarding the effect of the fiber on healthy cells, tests were performed on ATCC PCS-600-010 for 48 h. The results showed that cell proliferation and viability at a concentration of 50 μg/mL were about 4% lower than those of the control, suggesting that the Rh-MOF/PVA-PVP nanofiber has no adverse effects on healthy cells.

Therefore, as expected, the Rh-MOF/PVA-PVP nanofiber exhibited significant anticancer properties. Its ability to act against skin cancer cells and a wide range of bacterial skin infections indicates the effective composition of the Rh-MOF/PVA-PVP nanofiber synthesized in this study. After examining other factors affecting the industrial use of the Rh-MOF/PVA-PVP nanofiber as wound dressings and bandages, including *in vivo* tests, it can be introduced into the expansion of this field.

## 4 Conclusion

Nanofibers based on compounds containing biological materials such as rhodium and 2,2′-bipyridine-4,4′-dicarboxylic acid were synthesized (Rh-MOF/PVA-PVP nanofiber) using PVA and PVP (as an environmentally friendly polymer) through electrospinning. The presence of these compounds in the final product contributed to its effectiveness in inhibiting specialized bacterial strains associated with skin infections, precisely strains *S. pyogenes, S. dysgalactiae subsp. equisimilis*, and *S. anginosus*, *Staphylococcus aureus, Pseudomonas aeruginosa*, and *Corynebacterium minutissimum*, as well as skin cancer cells. Importantly, no significant adverse effects were observed against healthy skin cells. The high efficacy of the synthesized Rh-MOF/PVA-PVP nanofiber in this study can be attributed to the incorporating of compounds with notable biological properties and specific structural features, such as a high specific surface area, which enhances contact between the fiber and pathogens. This specific active surface area is characteristic of nanocompounds and, along with other attributes like crystallinity and nanoscale dimensions, is influenced by the synthesis method. The results obtained in this study confirmed the appropriateness of the synthesis method employed. Finally, by conducting additional biological tests, such as *in vitro* studies, the synthesized Rh-MOF/PVA-PVP nanofiber can be introduced into the medical industry for use in wound dressings and bandages. Furthermore, the method used for synthesizing Rh-MOF/PVA-PVP nanofiber can be recommended for developing similar fiber compounds.

## Data Availability

The original contributions presented in the study are included in the article/supplementary material, further inquiries can be directed to the corresponding author.
